# Supplementary material for: The Ecology and Phylogeny of Hosts Drive the Enzootic Infection Cycles of Hantaviruses
Source: Viruses. 2019 Jul 23;11(7):671. doi: 10.3390/v11070671 (PMC6669546; doi:10.3390/v11070671)
Supplement: Supplementary file 1 [file viruses-11-00671-s001.zip › MILHOLLAND_ET_AL_VIRUSES_2019_S3.pdf]

S3. Rodent hosts of *Orthohantaviruses* (Order: Bunyavirales; Family: Hantaviridae) from assemblages reported in literature published between 1971-2014. Species are arranged alphabetically and in decreasing taxonomic hierarchy. Infection were confirmed by RT-PCR or antibody detection assays. (Wilson, D.E.; Reeder, D.M. Mammal species of the world a taxonomic and geographic reference, 3rd ed.; The Johns Hopkins University Press: Baltimore, MD, USA, 2005).

Suborder	Family	Subfamily	Genus	Species	Sites	N_{tot}	N_{inf}
CASTORIMORPHA	*Geomyidae*		*Geomys*	*Geomys bursarius*	1	1	0
			Thomomys	*Thomomys bottae*	2	14	0
	Heteromyidae	*Dipodomyinae*	*Dipodomys*	*Dipodomys microps*	1	11	0
				Dipodomys ordii	13	108	0
				Dipodomys spectabilis	1	1	0
		Heteromyinae	*Heteromys*	*Heteromys anomalus*	4	18	1
			Liomys	*Liomys irroratus*	1	12	1
				Liomys pictus	1	18	0
				Liomys salvini	1	2	0
				Liomys spectabilis	1	6	0
		Perognathinae	*Chaetodipus*	*Chaetodipus baileyi*	1	1	0
				Chaetodipus californicus	2	4	0
				Chaetodipus eremicus	1	18	0
				Chaetodipus hispidus	10	28	0
				Chaetodipus intermedius	1	13	0
				Chaetodipus penicillatus	3	33	0
			Perognathus	*Perognathus fasciatus*	1	2	1
				Perognathus flavescens	1	2	0
				Perognathus flavus	5	28	0
				Perognathus parvus	8	125	1
HYSTRICOMORPHA	*Echimyidae*	*Eumysopinae*	*Proechimys*	*Proechimys canicollis*	1	2	1
				Proechimys guairae	2	3	0
				Proechimys guyannensis	1	1	0
MYOMORPHA	*Cricetidae*	*Arvicolinae*	*Eothenomys*	*Eothenomys miletus*	1	129	23
			Lemmiscus	*Lemmiscus curtatus*	6	35	0

		Microtus	*Microtus agrestis*	8	109	8
			Microtus arvalis	6	126	17
			Microtus californicus	1	6	1
			Microtus fortis	2	25	3
			Microtus longicaudus	5	24	0
			Microtus mexicanus	7	41	3
			Microtus montanus	14	59	1
			Microtus ochrogaster	4	11	1
			Microtus pennsylvanicus	6	230	60
			Microtus pinetorum	1	2	0
			Microtus subterraneus	2	3	0
		Myodes	*Myodes gapperi*	9	18	2
			Myodes glareoulus	15	571	77
			Myodes rufocanus	4	142	24
MYOMORPHA	*Neotominae*	*Baiomys*	*Baiomys musculus*	5	84	2
			Baiomys taylori	11	185	2
		Megadontomys	*Megadontomys thomasi*	2	7	1
		Neotoma	*Neotoma albigula*	13	156	2
			Neotoma cinerea	5	16	0
			Neotoma floridana	4	15	0
			Neotoma lepida	6	75	0
			Neotoma leucodon	2	20	0
			Neotoma mexicana	12	40	0
			Neotoma micropus	4	42	0
			Neotoma picta	2	8	1
			Neotoma stephensi	2	8	0
		Neotomodon	*Neotomodon alstoni*	2	3	0
		Ochrotomys	*Ochrotomys nuttalli*	2	2	0
		Onychomys	*Onychomys arenicola*	2	3	0
			Onychomys leucogaster	8	28	1

		Onychomys torridus	1	1	0
	Osgoodomys	*Osgoodomys banderanus*	2	11	0
	Peromyscus	*Peromyscus attwateri*	4	181	9
		Peromyscus aztecus	1	4	0
		Peromyscus beatae	3	59	17
		Peromyscus boylii	18	359	72
		Peromyscus californicus	1	6	0
		Peromyscus difficilus	3	12	0
		Peromyscus eremicus	4	24	1
		Peromyscus gossypinus	2	6	2
		Peromyscus hylocytes	3	29	4
		Peromyscus lepturus	1	5	0
		Peromyscus leucopus	38	581	50
		Peromyscus levipes	5	72	11
MYOMORPHA		*Peromyscus maniculatus*	78	3571	460
		Peromyscus megalops	2	53	1
		Peromyscus melanophrys	3	18	0
		Peromyscus melanotis	6	78	5
		Peromyscus mexicanus	3	12	0
		Peromyscus nasutus	3	4	1
	Peromyscus	*Peromyscus ochraventer*	2	23	2
		Peromyscus pectoralis	2	8	1
		Peromyscus perfulvus	1	16	0
		Peromyscus spicilegus	1	9	2
		Peromyscus truei	22	138	4
	Reithrodontomys	*Reithrodontomys bakeri*	1	3	0
		Reithrodontomys fulvescens	23	492	2
		Reithrodontomys humulis	2	3	0
		Reithrodontomys megalotis	25	145	20
		Reithrodontomys mexicanus	3	20	0

			Reithrodontomys microdon	1	1	1
			Reithrodontomys montanus	2	3	0
			Reithrodontomys sumichrasti	4	35	12
	Sigmodontinae	*Abrothrix*	*Abrothrix longipilis*	3	92	4
			Abrothrix olivaceus	2	84	6
		Akodon	*Akodon aliquantulus*	1	3	0
			Akodon azarae	6	304	27
			Akodon cursor	3	33	2
			Akodon montensis	3	30	2
			Akodon simulator	1	140	4
			Akodon toba	2	5	1
		Calomys	*Calomys callosus*	5	179	10
			Calomys laucha	3	23	5
			Calomys musculinus	2	6	0
			Calomys tener	3	17	0
MYOMORPHA		*Graomys*	*Graomys griseoflavus*	1	2	0
		Holochilus	*Holochilus chacarius*	1	11	0
			Holochilus sciureus	2	52	15
		Necromys	*Necromys lasiurus*	11	293	18
			Necromys lenguarum	1	2	0
			Necromys obscurus	1	1	0
		Nectomys	*Nectomys squamipes*	4	7	0
		Oligoryzomys	*Oligoryzomys chacoensis*	1	4	0
			Oligoryzomys flavescens	9	64	5
			Oligoryzomys fornesi	3	14	7
			Oligoryzomys fulvescens	3	3	2
			Oligoryzomys griSeoulus	2	7	1
			Oligoryzomys longicaudatus	5	164	20
			Oligoryzomys microtis	3	96	13
		Oligoryzomys	*Oligoryzomys nigripes*	11	116	16

			Oryzomys	*Oryzomys alfaroi*	1	1	0
				Oryzomys chacoensis	1	1	0
				Oryzomys chapmani	1	4	0
				Oryzomys couesi	4	419	33
				Oryzomys melanotis	1	4	0
				Oryzomys mexicanus	1	3	0
				Oryzomys palustris	17	942	140
				Oryzomys ratticeps	1	4	0
			Oxymycterus	*Oxymycterus inca*	2	9	0
				Oxymycterus judex	1	5	0
				Oxymycterus nasutus	1	89	4
				Oxymycterus paramensis	1	3	0
			Pseudoryzomys	*Pseudoryzomys simplex*	1	1	0
			Scapteromys	*Scapteromys tumidus*	1	19	0
MYOMORPHA			*Sigmodon*	*Sigmodon alleni*	1	1	0
				Sigmodon alstoni	9	315	45
				Sigmodon arizonae	1	9	0
				Sigmodon hirsutus	1	37	0
				Sigmodon hispidus	33	1863	75
				Sigmodon macotensis	2	97	6
				Sigmodon toltecus	2	2	0
			Thaptomys	*Thaptomys nigrita*	1	2	1
			Zygodontomys	*Zygodontomys brevicauda*	10	145	5
		Tylomyinae	*Nyctomys*	*Nyctomys sumichrasti*	1	1	0
	Dipodidae	*Zapodinae*	*Zapus*	*Zapus princeps*	5	26	0
	Muridae	*Murinae*	*Apodemus*	*Apodemus agrarius*	13	460	19
				Apodemus argenteus	3	46	0
				Apodemus chevrieri	1	31	0
				Apodemus draco	1	19	0
				Apodemus flavicollis	12	309	68

				Apodemus peninsulae	1	70	4
				Apodemus speciosus	3	26	0
				Apodemus sylvaticus	10	188	10
				Apodemus uralensis	2	6	1
			Bandicota	*Bandicota indica*	13	360	18
				Bandicota savilei	3	122	5
			Berylmys	*Berylmys berdmorei*	5	34	0
				Berylmys bowersi	2	6	0
			Maxomys	*Maxomys surifer*	3	108	2
			Mus	*Mus caroli*	6	90	2
				Mus cervicolor	2	45	0
				Mus cookii	3	107	1
MYOMORPHA				*Mus musculus*	30	400	13
			Niviventer	*Niviventer confucianus*	2	66	7
				Niviventer fulvescens	3	17	0
			Rattus	*Rattus andamanensis*	1	5	0
				Rattus argentiventer	6	81	1
				Rattus exulans	11	537	16
				Rattus flavipectus	2	50	0
				Rattus fulvescens	1	2	0
				Rattus losea	8	281	4
				Rattus nitidus	3	42	4
				Rattus norvegicus	36	2424	502
				Rattus rattus	26	677	46
				Rattus tanezumi	13	524	19
	Sciuridae	*Callosciurinae*	*Callosciurus*	*Callosciurus erythraeus*	1	1	0
		Sciurinae	*Tamiasciurus*	*Tamiasciurus douglasii*	1	1	0
SCIUROMORPHA				*Tamiasciurus hudsonicus*	5	17	0
			Sciurus	*Siurus aberti*	1	4	0
		Xerinae	*Ammospermophilus*	*Ammospermophilus leucurus*	1	2	0

		Ammospermophilus harrisii	1	1	0
	Callospermophilus	*Callospermophilus lateralis*	4	14	0
	Eutamias	*Eutamias sibiricus*	1	2	0
	Neotamias	*Neotamias amoenus*	1	5	0
		Neotamias canipes	2	5	0
		Neotamias dorsalis	6	56	0
		Neotamias minimus	12	52	2
SCIUROMORPHA		*Neotamias quadrimaculatus*	1	7	0
		Neotamias quadrivittatus	8	34	0
		Neotamias rufus	1	3	0
		Neotamias speciosus	1	8	0
		Neotamias umbrinus	3	36	0
	Tamias	*Tamias striatus*	4	5	0
	Urocitellus	*Urocitellus armatus*	2	2	0
	Xerospermophilus	*Xerospermophilus spilosoma*	1	3	0
		Xerospermophilus variegatus	9	36	0
