# Supplementary material for: The Ecology and Phylogeny of Hosts Drive the Enzootic Infection Cycles of Hantaviruses
Source: Viruses. 2019 Jul 23;11(7):671. doi: 10.3390/v11070671 (PMC6669546; doi:10.3390/v11070671)
Supplement: Supplementary file 1 [file viruses-11-00671-s001.zip › MILHOLLAND_ET_AL_VIRUSES_2019_S1.pdf]

North American Assemblage Locality Citations:

1. Arellano, E.; Castro-Arellano, I.; Suzán, G.; González-Cózatl, F.X.; Jiménez, R.M. Antibody seroprevalence to hantaviruses in rodents from Reserva De La Biosfera Sierra De Huautla, Morelos. *West. North Am. Nat.* **2012**, *72*, 105–109.
2. Baek, L.J.; Yanagihara, R.; Gibbs, C.J.; Miyazaki, M.; Gajdusek, D.C. Leakey virus: a new hantavirus isolated from *Mus musculus* in the United States. *J. Gen. Virol.* **1988**, *69*, 3129–3132.
3. Castro-Arellano, I.; Suzán, G.; León, R.F.; Jiménez, R.M.; Lacher, T.E. Survey for antibody to hantaviruses in Tamaulipas, Mexico. *J. Wildl. Dis.* **2009**, *45*, 1, 207-212.
4. Chu, Y.K.; Owen, R.D.; Sánchez-Hernández, C.; Romero-Almaraz, M. de L.; Jonsson, C.B. Genetic characterization and phylogeny of a hantavirus from western Mexico. *Virus Res.* **2008**, *131*, 180–188.
5. Clay, C.A.; Lehmer, E.M.; Jeor, S.S.; Dearing, M.D. Testing mechanisms of the dilution effect: deer mice encounter rates, Sin Nombre virus prevalence and species diversity. *Ecohealth* **2009**, *6*, 250–259.
6. Hjelle, B.; Lee, S.; Song, W. Molecular linkage of hantavirus pulmonary syndrome to the white-footed mouse, *Peromyscus leucopus*: genetic characterization of the M genome of New York virus. *J. Virol.* **1995**, *69*, 8137–8141.
7. Hjelle, B.; Torrez-Martinez, N.; Koster, F.T.; Jay, M.; Ascher, M.S.; Brown, T.; Reynolds, P.; Ettestad, P.; Voorhees, R.E.; Sarisky, J.; et al. Epidemiologic linkage of rodent and human hantavirus genomic sequences in case investigations of hantavirus pulmonary syndrome. *J. Infect. Dis.* **1996**, *173*, 781–786.
8. Jay, M.; Hjelle, B.; Davis, R.; Ascher, M.; Baylies, H.N.; Reilly, K.; Vugia, D. Occupational exposure leading to hantavirus pulmonary syndrome in a utility company employee. *Clin. Infect. Dis.* **1996**, *22*, 841–844.
9. Kariwa, H.; Yoshida, H.; Sánchez-Hernández, C.; Romero-Almaraz, M. de L.; Almazán-Catalán, J.A.; Ramos, C.; Miyashita, D.; Seto, T.; Takano, A.; Totani, M.; et al. Genetic diversity of hantaviruses in Mexico: Identification of three novel hantaviruses from Neotominae rodents. *Virus Res.* **2012**, *163*, 486–494.
10. Khan, A.S.; Khabbaz, R.F.; Armstrong, L.R.; Holman, R.C.; Bauer, S.P.; Graber, J.; Strine, T.; Miller, G.; Reef, S.; Tappero, J.; et al. Hantavirus pulmonary syndrome: The first 100 US cases. *J. Infect. Dis.* **1996**, *173*, 1297–1303.
11. Lee, P-W.; Amyx, H.L.; Yanagihara, R.; Gajdusek, D.C.; Goldgaber, D.; Gibbs Jr., C.J. Partial characterization of Prospect Hill virus isolated from meadow voles in the United States. *J. Infect. Dis.* **1985**, *152*, 4, 826-829.
12. Lonner, B.N.; Douglass, R.J.; Kuenzi, A.J.; Hughes, K. Seroprevalence against Sin Nombre Virus in resident and dispersing deer mice. *Vector-Borne Zoonotic Dis.* **2008**, *8*, 433–442.
13. Mantooth, S.J.; Milazzo, M.L.; Bradley, R.D.; Hice, C.L.; Ceballos, G.; Tesh, R.B.; Fulhorst, C.F. Geographical distribution of rodent-associated hantaviruses in

Texas. *J. vector Ecol.* **2001**, *26*, 7–14.
14. McIntyre, N.E.; Chu, Y.K.; Owen, R.D.; Abuzeineh, A.; De La Sancha, N.; Dick, C.W.; Holsomback, T.; Nisbett, R.A.; Jonsson, C. A longitudinal study of Bayou virus, hosts, and habitat. *Am. J. Trop. Med. Hyg.* **2005**, *73*, 1043–1049.
15. Milazzo, M.L.; Cajimat, M.N.B.; Romo, H.E.; Estrada-Franco, J.G.; Ignacio Iñiguez-Dávalos, L.; Bradley, R.D.; Fulhorst, C.F. Geographic distribution of hantaviruses associated with neotomine and sigmodontine rodents, Mexico. *Emerg. Infect. Dis.* **2012**, *18*, 571–576.
16. Milazzo, M.L.; Cajimat, M.N.B.; Duno, G.; Duno, F.; Utrera, A.; Fulhorst, C.F. Transmission of guanarito and pirital viruses among wild rodents, Venezuela. *Emerg. Infect. Dis.* **2011**, *17*, 2209–2215.
17. Mills, J.N.; Ksiazek, T.G.; Ellis, B.A.; Rollin, P.E.; Nichol, S.T.; Yates, T.L.; Gannon, W.L.; Levy, C.E.; Engelthaler, D.M.; Davis, T.; et al. Patterns of association with host and habitat: Antibody reactive with Sin Nombre virus in small mammals in the major biotic communities of the southwestern United States. *Am. J. Trop. Med. Hyg.* **1997**, *56*, 273–284.
18. Childs, J.E.; Yates, T.L.; Miyashiro, J.; Zyzak, M.; Patrick, M.; Rollin, P.E.; Ksiazek, T.G.; Mann, M.O.; Johnson, M.R.; Novak, M.G.; et al. A survey of hantavirus antibody in small-mammal populations in selected United States National Parks. *Am. J. Trop. Med. Hyg.* **2017**, *58*, 525–532.
19. Nisbett, R. a; Caire, W.; Stuart, M.D.; Caddell, G.M.; Crutcher, J.M.; Calisher, C.H. Serologic survey of Oklahoma rodents: evidence for the presence of a hantavirus and an arenavirus. *Proc. Oklahoma Acad. Sci.* **2001**, *81*, 53–66.
20. Rhodes, L. V.; Huang, C.; Sanchez, A.J.; Nichol, S.T.; Zaki, S.R.; Ksiazek, T.G.; Humphreys, J.G.; Freeman, J.J.; Knecht, K.R. Hantavirus pulmonary syndrome associated with Monongahela virus, Pennsylvania. *Emerg. Infect. Dis.* **2000**, *6*, 616–621.
21. Suzán, G.; Ceballos, G.; Mills, J.; Ksiazek, T.G.; Yates, T. Serologic Evidence of Hantavirus Infection in Sigmodontine Rodents in Mexico. *J. Wildl. Dis.* **2013**, *37*, 391–393.
22. Torrez-Martinez, N.; Bharadwaj, M.; Goade, D.; Delury, J.; Moran, P.; Hicks, B.; Nix, B.; Davis, J.L.; Hjelle, B. Bayou virus-associated hantavirus pulmonary syndrome in eastern Texas: Identification of the rice rat, *Oryzomys palustris*, as reservoir host. *Emerg. Infect. Dis.* **1998**, *4*, 105–111.
23. Turell, M.J.; Korch, G.W.; Rossi, C.A.; Sesline, D.; Enge, B.A.; Dondero, D.V.; Jay, M.; Ludwig, G.V.; Li, D.; Schmaljohn, C.S.; Jackson, R.J.; Ascher, M.S. Short report: prevalence of hantavirus infection in rodents associated with two fatal human infections in California. *Am. J. Trop. Med. Hyg.* **1995** *52*, 2, 180-182.

South American Assemblage Locality Citations:

24. Alemán, A.; Iguarán, H.; Puerta, H.; Cantillo, C.; Mills, J.; Ariz, W.; Mattar, S. Primera evidencia serológica de infección por Hantavirus en roedores, en Colombia. *Rev. Salud Pública* **2006**, *8*, 1–12.
25. Bayard, V.; Kitsutani, P.T.; Barria, E.O.; Ruedas, L.A.; Tinnin, D.S.; Muñoz, C.; de Mosca, I.B.; Guerrero, G.; Kant, R.; Garcia, A.; et al. Outbreak of hantavirus pulomonary syndrome, Los Santos, Panama, 1999-2000. *Emerg. Infect. Dis.* **2004**, *10*, 9, 1635-1642.
26. Carroll, D.S.; Mills, J.N.; Montgomery, J.M.; Bausch, D.G.; Blair, P.J.; Burans, J.P.; Felices, V.; Gianella, A.; Iihoshi, N.; Nichol, S.T.; et al. Hantavirus pulmonary syndrome in central Bolivia: relationships between reservoir hosts, habitats, and viral genotypes. *Am. J. Trop. Med. Hyg.* **2005**, *72*, 1, 42–46.
27. De Araujo, J.; Thomazelli, L.M.; Henriques, D.A.; Lautenschalager, D.; Ometto, T.; Dutra, L.M.; Aires, C.C.; Favorito, S.; Durigon, E.L. Detection of hantavirus in bats from remaining rain forest in São Paulo, Brazil. *BMC Res. Notes* **2012**, *5*, 1.
28. De Sousa, R.L.M.; Moreli, M.L.; Borges, A.A.; Campos, G.M.; Livonesi, M.C.; Figueiredo, L.T.M.; Pinto, A.A. Natural host relationships and genetic diversity of rodent-associated hantaviruses in Southeastern Brazil. *Intervirology* **2008**, *51*, 299–310.
29. Delfraro, A.; Tomé, L.; D'Elía, G.; Clara, M.; Achával, F.; Russi, J.C.; Arbiza Rodonz, J.R. Juquitiba-like hantavirus from 2 nonrelated rodent species, Uruguay. *Emerg. Infect. Dis.* **2008**, *14*, 1447–1451.
30. Milazzo, M.L.; Cajimat, M.N.B.; Hanson, J.D.; Bradley, R.D.; Quintana, M.; Sherman, C.; Velásquez; Fulhorst, C.F. Catacamas virus, a hantaviral species naturally associated with *Oryzomys couesi* (Coues' *Oryzomys*) in Honduras. *Am. J. Trop. Med. Hyg.* **2006**, *75*, 5, 1003-1010.
31. Milazzo, M.L.; Duno, G.; Utrera, A.; Richter, M.H.; Duno, F.; de Manzione, N.; Fulhorst, C.F. Natural Host Relationships of Hantaviruses Native to Western Venezuela. *Vector-Borne Zoonotic Dis.* **2010**, *10*, 605–611.
32. Padula, P.; Martinez, V.P.; Bellomo, C.; Maidana, S.; Juan, J.S.; Tagliaferri, P.; Bargardi, S.; Vazquez, C.; Colucci, N.; Estévez, J. Pathogenic Eastern Paraguay. *Emerg. Infect. Dis.* **2007**, *13*, 1211–1214.
33. Pini, N.; Levis, S.; Calderón, G.; Ramirez, J.; Bravo, D.; Lozano, E.; Ripoll, C.; St. Jeor, S.; Ksiazek, T.G.; Barrquez, R.M.; Enria, D. Hantavirus infection in humans and rodents, northwestern Argentina. *Emerg. Infect. Dis.* **2003**, *9*, 9, 1070-1076.
34. Polop, F.J.; Provensal, M.C.; Pini, N.; Levis, S.C.; Priotto, J.W.; Enría, D.; Calderón, G.E.; Costa, F.; Polop, J.J. Temporal and spatial host abundance and prevalence of Andes hantavirus in Southern Argentina. *Ecohealth* **2010**, *7*, 176–184.
35. Piudo, L.; Monteverde, M.; Capria, S.G.; Padula, P.; Carmanchahi, P. Distribution and abundance of sigmodontine rodents in relation to hantavirus in Neuquén,

Argentina. *J. Vector Ecol.* **2005**, *30*, 119–125.
36. Powers, A.M.; Mercer, D.R.; Watts, D.M.; Guzman, H.; Fulhorst, C.F.; Popov, V.L.; Tesh, R.B. Isolation and genetic characterization of a hantavirus (Bunyaviridae: Hantavirus) from a rodent, *Oligoryzomys microtis* (Muridae), collected in northeastern Peru. *Am. J. Trop. Med. Hyg.* **1999**, *61*, 92–98.
37. Rosa, E.S.T.; Mills, J.N.; Padula, P.J.; Elkhoury, M.R.; Ksiazek, T.G.; Mendes, W.S.; Santos, E.D.; Araújo, G.C.B.; Martinez, V.P.; Rosa, J.F.S.T.; et al. Newly recognized hantaviruses associated with hantavirus pulmonary syndrome in northern Brazil: partial genetic characterization of viruses and serologic implication of likely reservoirs. *Vector-Borne Zoonotic Dis.* **2005**, *5*, 11–19.
38. Sobreira, M.; Souza, G.T.; Moreli, M.L.; Borges, A.A.; Morais, F.A.; Figueiredo, L.T.M.; Almeida, A.M.P. A serosurvey for hantavirus infection in wild rodents from the states of Rio de Janeiro and Pernambuco, Brazil. *Acta Trop.* **2008**, *107*, 150–152.
39. Suárez, O. V.; Cueto, G.R.; Cavia, R.; Villafañe, I.E.G.; Bilenca, D.N.; Edelstein, A.; Martínez, P.; Miguel, S.; Bellomo, C.; Hodara, K.; et al. Prevalence of infection with hantavirus in rodent populations of central Argentina. *Mem. Inst. Oswaldo Cruz* **2003**, *98*, 727–732.
40. Suzuki, A.; Bisordi, I.; Levis, S.; Garcia, J.; Pereira, L.E.; Sousa, R.P.; Sugahara, T.K.N.; Pini, N.; Enria, D.; Souza, L.T.M. Identifying rodent hantavirus reservoirs, Brazil. *Emerg. Infect. Dis.* **2004**, *10*, 2127–2134.
41. Toro, J.; Vega, J.D.; Khan, A.S.; Mills, J.N.; Terry, W.; Yadón, Z.; Valderrama, R.; Barbara, A.; Pavletic, C.; Cerda, R.; et al. An outbreak of hantavirus pulmonary syndrome, Chile, 1997. *Emerg Infect Dis* **1998**, *4*, 687–694.
42. da Rosa, E.S.T.; de Lemos, E.R.S.; Medeiros, D.B. D. A.; Simith, D.B.; Pereira, A. de S.; Elkhoury, M.R.; Mendes, W.S.; Vidigal, J.R.B.; de Oliveira, R.C.; D'Andrea, P.S.; et al. Hantaviruses and hantavirus pulmonary syndrome, Maranhão, Brazil. *Emerg. Infect. Dis.* **2010**, *16*, 1952–1955.
43. Williams, R.J.; Bryan, R.T.; Mills, J.N.; Palma, R.E.; Vera, I.; De Velasquez, F.; Baez, E.; Schmidt, W.E.; Figueroa, R.E.; Peters, C.J.; et al. An outbreak of hantavirus pulmonary syndrome in western Paraguay. *Am. J. Trop. Med. Hyg.* **1997**, *57*, 274–282.

Asian Assembalge Locality Citations:

44. Arai, S.; Bennett, S.N.; Sumibcay, L.; Cook, J.A.; Song, J.W.; Hope, A.; Parmenter, C.; Nerurkar, V.R.; Yates, T.L.; Yanagihara, R. Short report: phylogenetically distinct hantaviruses in the masked shrew (*Sorex cinereus*) and dusky shrew (*Sorex monticolus*) in the United States. *Am. J. Trop. Med. Hyg.* **2008**, *78*, 348–351.
45. Blasdell, K.; Cosson, J.F.; Chaval, Y.; Herbreteau, V.; Douangboupha, B.; Jittapalapong, S.; Lundqvist, A.; Hugot, J.P.; Morand, S.; Buchy, P. Rodent-borne

hantaviruses in Cambodia, Lao PDR, and Thailand. *Ecohealth* **2011**, *8*, 432–443.
46. Chin, C.; Chiueh, T.S.; Yang, W.C.; Yang, T.H.; Shih, C.M.; Lin, H.T.; Lin, K.C.; Lien, J.C.; Tsai, T.F.; Ruo, S.L.; et al. Hantavirus infection in Taiwan: the experience of a geographically unique area. *J. Med. Virol.* **2000**, *60*, 237–247.
47. Huong, V.T.Q.; Yoshimatsu, K.; Luan, V.D.; Tuan, L.V.; Nhi, L.; Arikawa, J.; Nguyen, T.M.N. Hemorrhagic fever with renal syndrome, Vietnam. *Emer. Infect. Dis.* **2010**, *16*, 2, 363-365.
48. Ibrahim. I-N.; Shimizu, K.; Yoshimatsu, K.; Yunianto, A.; Salwati, E.; Yasuda, S. P.; Koma, T.; Endo, R.; Arikawa, J. Epidemiology of hantavirus infection in Thousand Islands Regency of Jakarta, Indonesia. *J. Vet. Med. Sci.* **2013**, JVMS_12-0442.
49. Kariwa, H.; Yoshimatsu, K.; Sawabe, J.; Yokota, E.; Arikawa, J.; Takashima, I.; Fukushima, H.; Lundkvist, Å.; Shubin, F.N.; Isachkova, L.M.; et al. Genetic diversities of hantaviruses among rodents in Hokkaido, Japan and far east Russia. *Virus Res.* **1999**, *59*, 219–228.
50. Lin, X.-D.; Wang, W.; Guo, W.-P.; Zhang, X.-H.; Xing, J.-G.; Chen, S.-Z.; Li, M.-H.; Chen, Y.; Xu, J.; Plyusnin, A.; et al. Cross-Species Transmission in the Speciation of the Currently Known Murinae-Associated Hantaviruses. *J. Virol.* **2012**, *86*, 11171–11182.
51. Lokugamage, K.; Kariwa, H.; Hayasaka, D.; Cui, B.Z.; Iwasaki, T.; Lokugamage, N.; Ivanov, L.I.; Volkov, V.I.; Demenev, V.A.; Slonova, R.; et al. Genetic characterization of hantaviruses transmitted by the Korean field mouse (*Apodemus peninsulae*), Far East Russia. *Emerg. Infect. Dis.* **2002**, *8*, 768–776.
52. Luan, V.D.; Yoshimatsu, K.; Endo, R.; Taruishi, M.; Huong, V.T.; Dat, D.T.; Tien, P.C.; Shimizu, K.; Koma, T.; Yasuda, S.P.; et al. Studies on hantavirus infection in small mammals captured in southern and central highland area of Vietnam. *J. Vet. Med. Sci.* **2012**, *74*, 1155–1162.
53. Wong, T.W.; Chan, Y.C.; Joo, Y.G.; Lee, H.W.; Lee, P, W.; Yanagihara, R. Hantavirus infections in human and commensal rodents in Singpore. *Trans. Roy. Soc. Trop. Med. Hyg.* **1989**, *83*, 248-251.
54. Xu, Z.Y.; Tang, Y.W.; Kan, L.Y.; Tsai, T.F. Cats-source of protection or infection? A case-control study of hemorrhagic fever with renal syndrome. *Am. J. Epidemiol.* **1987** *126*, 5, 942-948.
55. Zhang, Y.; Yuan, J.; Yang, X.; Zhou, J.; Yang, W.; Peng, C.; Zhang, H.L.; Shi, Z. A novel hantavirus detected in Yunnan red-backed vole (*Eothenomys miletus*) in China. *J. Gen. Virol.* **2011**, *92*, 1454–1457.
56. Zhang, Y.Z.; Dong, X.; Li, X.; Ma, C.; Xiong, H.P.; Yan, G.J.; Gao, N.; Jiang, D.M.; Li, M.H.; Li, L.P.; et al. Seoul virus and hantavirus disease, Shenyang, People's Republic of China. *Emerg. Infect. Dis.* **2009**, *15*, 200–206.

European Assemblage Locality Citations:

57. Essbauer, S.S.; Schidt-Chanasit, J.; Madeja, E.L.; Wegener, W.; Friedrich, R.; Petraityte, R.; Sasnauskas, K.; Jacob, J.; Koch, J.; Dobler, G.; et al. Nephropathia epidemica in metropolitan area, Germany. *Emerg. Infect. Dis.* **2007**, *13*, 8, 1271-1273.
58. Gligic, A.; Dimkovic, N.; Xiao, S.Y.; Buckle, G.J.; Jovanovic, D.; Velimirovic, D.; Stojanovic, R.; Obradovic, M.; Diglisic, G.; Micic, J.; et al. Belgrade virus: a new hantavirus causing severe hemorrhagic fever with renal syndrome in yugoslavia. *J. Infect. Dis.* **1992**, *166*, 113–120.
59. Horling, J.; Chizhikov, V.; Lundkvist, Å.; Jonsson, M.; Ivanov, L.; Dekonenko, A.; Niklasson, B.; Dzagurova, T.; Peters, C.J.; Tkachenko, E.; et al. Khabarovsk virus: a phylogenetically and serologically distinct hantavirus isolated from *Microtus fortis* trapped in far-east Russia. *J. Gen. Virol.* **1996**, *77*, 687–694.
60. Taori, S.K.; Jameson, L.J.; Campbell, A.; Drew, P.J.; McCarthy, N.D.; Hart, J.; Osborne, J.C.; Sudhanva, M.; Brooks, T.J. UK hantavirus, renal failure, and pet rats. *Lancet* **2013**, *381*, 1070.
61. Jameson, L.J.; Logue, C.H.; Atkinson, B.; Baker, N.; Galbraith, S.E.; Carroll, M.W.; Brooks, T.; Hewson, R. The continued emergence of hantaviruses : isolation of a Seoul virus implicated in human disease , United Kingdom , Eurosurveillance.org, October 2012. **2013**, 1–4.
62. Scharninghausen, J.J.; Meyer, H.; Pfeffer, M.; Davis, D.S.; Honeycutt, R.L. Genetic evidence of Dobrava virus in *Apodemus agrarius* in Hungary. *Emerg. Infect. Dis.* **1999**, *5*, 468–470.
63. Scharninghausen, J.J.; Pfeffer, M.; Meyer, H.; Davis, D.S.; Honeycutt, R.L.; Faulde, M. Genetic evidence for Tula virus in *Microtus arvalis* and *Microtus agrestis* populations in Croatia. **2002**, *2*, 19–27.
64. Schmidt, S.; Essbauer, S.S.; Mayer-Scholl, A.; Poppert, S.; Schmidt-Chanasit, J.; Klempa, B.; Henning, K.; Schares, G.; Groschup, M.H.; Spitzenberger, F.; et al. Multiple infections of rodents with zoonotic pathogens in Austria. *Vector-Borne Zoonotic Dis.* **2014**, *14*, 467–475.
65. Song, J-W.; Baek, L.J.; Song, K-J, Skrok, A.; Markowski, J.; Bratosiewicz-Wasik, J.; Kordek, R.; Liberski, P.P.; Yanagihara, R. Characterization of Tula virus from common voles (*Microtus arvalis*) in Poland: evidence for geographic-specific phylogenetic clustering. *Virus Genes* **2004**, *29*, 2, 239-247.
66. Song, J.; Gu, S.H.; Bennett, S.N.; Arai, S.; Puorger, M.; Hilbe, M.; Yanagihara, R. common shrew (Sorex araneus). **2007**, *5*, 1–5.
67. Weidmann, M.; Schmidt, P.; Vackova, M.; Krivanec, K.; Hufert, F.T.; Identification of genetic evidence for Dobrava virus spillover in rodents by nested reverse transcription (RT)-PCR and TaqMan RT-PCR. *J. Clin. Microbiol.* **2005**, *43*, 2, 808.
