# Supplementary material for: The Ecology and Phylogeny of Hosts Drive the Enzootic Infection Cycles of Hantaviruses
Source: Viruses. 2019 Jul 23;11(7):671. doi: 10.3390/v11070671 (PMC6669546; doi:10.3390/v11070671)
Supplement: Supplementary file 1 [file viruses-11-00671-s001.zip › MILHOLLAND_ET_AL_VIRUSES_2019_S2.pdf]

S2. Non-rodent small mammal hosts of *Orthohantaviruses* (Order: Bunyavirales; Family: Hantaviridae) from assemblages reported in literature published between 1971-2014. Species are arranged alphabetically and in decreasing taxonomic hierarchy. Infection were confirmed by RT-PCR or antibody detection assays. Taxonomy adopted from (Wilson, D.E.; Reeder, D.M. *Mammal species of the world a taxonomic and geographic reference* , 3rd ed.; The Johns Hopkins University Press: Baltimore, MD, USA, 2005).

Order	Family	Subfamily	Genus	Species	Sites	N_{tot}	N_{inf}
CARNIVORA	*Mustelidae*	*Mustelinae*	*Mustela*	*Mustela frenata*	1	3	0
				Mustela nivalis	1	2	0
DIDELPHIMORPHIA	*Didelphidae*	*Didelphinae*	*Didelphis*	*Didelphis virginiana*	1	3	0
			Marmosa	*Marmosa murina*	1	1	0
				Marmosa paraguayana [1] *	1	3	1
			Marmospos	*Marmosops incanus*	1	2	0
				Marmosops paulensis	1	2	0
			Monodelphis	*Monodelphis americana* [2]	1	1	0
				*Monodelphis iheringi**	1	6	1
			Thylamys	*Thylamys elegans*	1	1	0
				Thylamys venustus	1	2	0
			Tlacuatzin	*Tlacuatzin canescens*	1	10	0
EULIPOTYPHLA	*Soricidae*	*Crocidurinae*	*Crocidura*	*Crocidura dsinezumi*	1	1	0
				Crocidura leucodon	1	3	0
				Crocidura suaveolens	2	13	0
			Suncus	*Suncus murinus**	20	1186	19
		Soricinae	*Blarina*	*Blarina brevicauda*	4	21	0
				Blarina hylophaga	2	3	0
			Blarinella	*Blarinella wardi*	1	7	0
			Cryptotis	*Cryptotis obscura*	1	1	0
				Cryptotis parva	1	1	0
			Neomys	*Neomys anomalus*	1	1	0
				Neomys fodiens	1	2	0
			Sorex	*Sorex araneus*	5	9	0
				Sorex caecutiens	1	3	0
				Sorex fumeus	1	1	0
				Sorex gracillimus	1	5	0
				Sorex sinalis	1	5	0
				Sorex unguiculatus	1	6	0
				Sorex vagrans	1	1	0
	Talpidae	*Talpinae*	*Urotrichus*	*Urotrichus talpoides*	1	6	3
LAGOMORPHA	*Leporidae*		*Sylvilagus*	*Sylvilagus audobonii*	3	5	0
				Sylvilagus floridanus	2	2	0
SCANDENTIA	*Tupaiidae*		*Tupaia*	*Tupaia ferruginea* [3]	1	3	0

[1] reported as *Micoureus paraguayanus* ; [2] reported as *Monodelphis rubida* ; [3] reported as *Tupaia glis* ; *infection confirmed with RT-PCR
